# Characterizing Safety and Clinical Outcomes Associated with High-Dose Micafungin Utilization in Patients with Proven Invasive Candidiasis

**DOI:** 10.3390/tropicalmed7020023

**Published:** 2022-02-03

**Authors:** Victoria C. Grant, Kenneth Nguyen, Sasha Rodriguez, Anna Y. Zhou, Jacinda C. Abdul-Mutakabbir, Karen K. Tan

**Affiliations:** 1 Department of Pharmacy Practice, Loma Linda University Medical Center, Loma Linda, CA 92354, USA; VCGrant@llu.edu (V.C.G.); AZhou@llu.edu (A.Y.Z.); 2 Department of Pharmacy Practice, Loma Linda University School of Pharmacy, Loma Linda, CA 92354, USA; kvnguyen@students.llu.edu (K.N.); SashaRodriguez@students.llu.edu (S.R.); JAbdulmutakabbir@llu.edu (J.C.A.-M.); 3 Department of Basic Sciences, Loma Linda University School of Medicine, Loma Linda, CA 92354, USA

**Keywords:** micafungin, invasive candidiasis, obesity, critically ill

## Abstract

Micafungin is the empiric antifungal agent of choice for the treatment of invasive candidiasis (IC). Pathophysiologic changes that occur in obese and/or critically ill patients can alter micafungin serum concentrations and the probability of target attainment. Although high doses of micafungin have been shown to be safe, clinical outcomes have not been widely evaluated. We conducted a single-center, retrospective observational study evaluating safety and clinical outcomes among adult patients treated with ≥200 mg of micafungin for ≥3 days for proven IC from 1 September 2013 through 1 September 2021. Twenty-three unique encounters for 21 patients were evaluated. The median BMI and APACHE II scores were 37.1 (IQR 28.8–48.9) and 24 (IQR 17.7–31), respectively. The median average daily dose of micafungin was 300 mg (IQR 275–400). Patients were treated with high-dose (HD) micafungin for the entirety of their echinocandin course in 15 encounters (65.2%). Transaminases remained stable, while a trend towards increased alkaline phosphatase was observed. A total of four deaths occurred (17.4%). Patients that died were predominantly young, Hispanic males who were obese and/or critically ill. Future studies are needed to determine the necessity and appropriate placement of HD micafungin in obese and/or critically ill patients.

## 1. Introduction

Invasive candidiasis (IC) is an increasing healthcare concern in the United States (US) and candidemia is one of the most common healthcare-associated bloodstream infections [1]. A recent, large, epidemiologic review evaluated 203 hospitals in the US from 2009 to 2017 and determined that 90 out of every 100,000 hospital encounters included a case of IC [2]. Among more than 16,000 patients with IC that were assessed, the mortality rate was 22%. In the subgroup of patients with candidemia, the rate of mortality was higher and reported as 28% [2]. Early detection and initiation of appropriate empiric antifungal therapy is crucial for the management of IC [3,4].

Echinocandins are the empiric antifungal agents of choice for the treatment of IC and are recommended as the first-line definitive therapy for infections due to select *Candida* species (i.e., *Candida krusei*) [5]. Micafungin is approved by the US Food and Drug Administration at doses of 100–150 mg (referred to as conventional doses throughout this paper) for the treatment of candidemia, IC (excluding meningoencephalitis, ocular dissemination, endocarditis, and osteomyelitis), and esophageal candidiasis [6]. However, recent studies have evaluated the pathophysiologic changes that occur in obese and/or critically ill patients and found that these changes may alter the pharmacokinetics of micafungin, subsequently altering serum concentrations and the probability of target attainment (PTA) [7]. It has previously been demonstrated that the area under the curve:minimum inhibitory concentration (AUC:MIC) ratio is the most appropriate pharmacodynamic target for micafungin [8]. 

A single-dose micafungin pharmacokinetic study in normal-weight (body mass index (BMI) 18.5–25 kg/m^2^) and morbidly obese (BMI > 40 kg/m^2^) healthy adults receiving 100 or 200 mg of micafungin was conducted [9]. Monte Carlo simulations were performed and determined that standard 100 mg doses of micafungin resulted in a PTA > 90% in patients ≤ 125 kg for *Candida* species with MICs ≤ 0.016 mg/L. Doses of up to 300 mg were required to achieve adequate target attainment among patients >125 kg and/or micafungin MICs > 0.016 mg/L. The authors also concluded that loading doses may be required to achieve adequate concentrations on day 1 of treatment for isolates with micafungin MICs ≥ 0.016 mg/L. A similar need for higher micafungin doses to achieve adequate target attainment has been demonstrated among critically ill patients [9].

The PTA on days 3 and 14 of micafungin among normal-weight (median BMI 24.6 kg/m^2^), critically ill adult patients that received micafungin 100 mg daily was estimated [10]. Monte Carlo simulations were performed using five different micafungin regimens. For a target AUC:MIC ratio of >3000 mcg ∗ hr/mL, a micafungin 200 mg loading dose followed by 150 mg daily was required to achieve >90% PTA on both days 3 and 14. Alternatively, for a target AUC:MIC ratio of >5000 mcg ∗ hr/mL, micafungin 200 mg daily was required to achieve a PTA of 86% and 90% on days 3 and 14, respectively [10]. 

Despite evidence suggesting that obese and/or critically ill patients exhibit altered micafungin pharmacokinetics leading to lower PTA, there is limited clinical data evaluating rates of treatment success/failure with administration of conventional doses of micafungin in these patient populations. Suboptimal serum micafungin concentrations leading to low PTA among these patients treated with conventional doses may increase the risk of therapeutic failure, and clinicians may need to consider higher micafungin doses for these patient populations. Micafungin has been shown to be safe and tolerable at doses up to 8 mg/kg/day [11]. However, clinical outcomes associated with high-dose (HD) regimens have not been widely evaluated. The objective of this study was to characterize safety and clinical outcomes associated with real-world, HD micafungin utilization in patients with proven IC.

## 2. Materials and Methods

### 2.1. Study Design, Patient Population, and Location

This was an Institutional Review Board-approved retrospective, observational study evaluating patients with proven IC at Loma Linda University Medical Center (LLUMC) and LLUMC East Campus from 1 September 2013 through 1 September 2021. Patients were included if they were ≥18 years old and received ≥200 mg of micafungin for ≥3 days. Patients were excluded if initial positive cultures were obtained at an outside institution prior to admission.

At LLUMC and LLUMC East Campus, micafungin is a restricted antimicrobial that requires approval by an infectious diseases (ID) attending physician. Currently, there are no specific indications or criteria for use regarding HD micafungin utilization at our institutions. The doses of micafungin used for the patients included in this study were determined by the ID attending physician on a case by case basis, but would often take into consideration patient weight and clinical status.

LLUMC and LLUMC East Campus are acute care teaching hospitals with approximately 450 licensed beds between both institutions. Both hospitals are located in San Bernardino county in Southern California, and LLUMC is the primary acute care and trauma institution that services the area. The county’s per capita income is among the lowest in the state and the population is predominantly Hispanic/Latino [12].

### 2.2. Study Definitions and Data Collection 

High-dose micafungin was defined as a dose ≥ 200 mg. As mentioned previously, micafungin doses of 100–150 mg are referred to as conventional doses. Proven IC was defined using the European Organization for Research and Treatment of Cancer and the Mycoses Study Group Education and Research Consortium (EORTC/MSGERC) consensus definitions of invasive fungal diseases [13].

Baseline demographics including age, gender, race/ethnicity, weight (kg), BMI (kg/m^2^), and comorbid conditions were collected. Patients were categorized as underweight, normal-weight, overweight, obese, or morbidly obese for a BMI of <18.5 kg/m^2^, 18.5 to <25 kg/m^2^, 25 to <30 kg/m^2^, 30 to <40 kg/m^2^, and ≥40 kg/m^2^, respectively [14]. Potential risk factors associated with the development of IC, including active malignancy, hematologic/solid organ transplant, intensive care unit (ICU) stay, use of broad-spectrum antibiotics, abdominal surgery within the previous 30 days, necrotizing pancreatitis, gastrointestinal (GI) perforation, anastomotic leak, receipt of total parenteral nutrition, and presence of a central line, were collected. Relevant clinical laboratory data were collected on the day of micafungin initiation to determine sepsis criteria and to calculate Acute Physiology and Chronic Health Evaluation (APACHE) II scores. APACHE II scores were only calculated for patients admitted to ICUs. Of note, sepsis criteria and APACHE II scores could not be determined for all patients due to missing data. Pertinent microbiology and treatment data were collected. Cultures were considered persistently positive if the same organism was isolated from the same site following the initiation of micafungin.

### 2.3. Outcomes of Interest

The primary safety outcomes were elevations in aspartate aminotransferase (AST), alanine aminotransferase (ALT), and alkaline phosphatase (ALP). Baseline AST, ALT, and ALP were collected within 24 h of initiation of HD micafungin. Post-HD micafungin AST, ALT, and ALP were collected within 24 h of discontinuation of HD micafungin. Aspartate aminotransferase, ALT, and ALP were considered to be within normal limits (WNL) for concentrations of 0–30 U/L, 7–37 U/L, and 37–132 U/L, respectively. Other commonly reported adverse reactions related to micafungin, including GI upset, headache, and thrombocytopenia, were evaluated. Incidence of rash, phlebitis, and/or infusion reaction was also collected.

The primary clinical outcome of interest was all-cause inpatient mortality. Total length of stay (LOS) and ICU LOS were also evaluated. All-cause 30-day readmission was collected. Recurrent infection, defined as isolation of the same organism from the same site following the completion of antifungal therapy, was determined.

### 2.4. Statistical Analysis

Study data were collected and managed using REDCap (version 10.5.1, Vanderbilt University, Nashville, TN, USA) electronic data capture tools hosted at LLUMC [15,16]. Data analyses were conducted on IBM SPSS version 28 (IBM, Armonk, NY, USA). Subgroup analyses were performed among patients dichotomized by their treatment course (entire cohort versus HD micafungin only) and end of hospital outcome (died versus survived). Continuous variables are reported as either mean or median based on the distribution. Categorical variables are reported as proportions.

## 3. Results

### 3.1. Baseline Demographics and Clinical Characteristics of the Study Population

Twenty-three unique encounters for 21 patients were evaluated. Table 1 includes select baseline demographics and clinical characteristics. The median age was 55 (IQR 34–66) years and patients were male in 17 encounters (73.9%). The median BMI was 37.1 (IQR 28.8–48.9) and patients were obese/morbidly obese in 16 (69.6%) encounters. Patients were predominantly Hispanic/Latino. The most common comorbid condition was diabetes mellitus and the most common risk factor for developing IC was the presence of an indwelling central line. 

Patients were in sepsis or septic shock at the time of micafungin initiation in 10 encounters (43.5%). Patients were admitted to the ICU at the time of micafungin initiation in 14 encounters (60.9%). Among ICU patients, the median APACHE II score was 24 (IQR 17.7–31).

### 3.2. Microbiology Data

Patients were candidemic in 10 encounters (43.5%). The most common source of infection was intra-abdominal (39.1%). Thirty-one Candida species were isolated from 25 cultures (Table 2). Six cultures were polymicrobial with two Candida species. Candida glabrata and Candida albicans were the most commonly isolated species. Micafungin MICs were available for 17 isolates (54.8%). Interpretations for the MICs were available for 15 isolates (15/17, 88.2%), all of which were susceptible to micafungin per Clinical Laboratory and Standards Institute M60 (June 2020)-defined breakpoints at the time of culture evaluation. Patients had persistently positive cultures in six encounters (26.1%).

### 3.3. Treatment Data

Patients were treated with a conventional dose of micafungin prior to the initiation of HD in three encounters (13%). Micafungin was reduced to a conventional dose following a course of HD in five encounters (21.7%). The median average daily dose (ADD) of micafungin for the entire cohort was 300 mg (IQR 275–400). This equated to a median weight-based dose of 2.84 mg/kg/day (IQR 2.4–3.9). The median duration of micafungin for the entire cohort was 11 days (IQR 6–16). Patients were treated with HD micafungin for the entirety of their echinocandin course in 15 encounters (65.2%). Among these patients, the median ADD, the median weight-based dose, and the median duration of micafungin were similar to those of the entire cohort. Table 3 displays treatment data among the entire cohort and among a subgroup of patients that received HD micafungin for the entirety of their echinocandin course. Source control was attempted in 21 encounters (91.3%).

### 3.4. Safety Data

**Figure 1:**
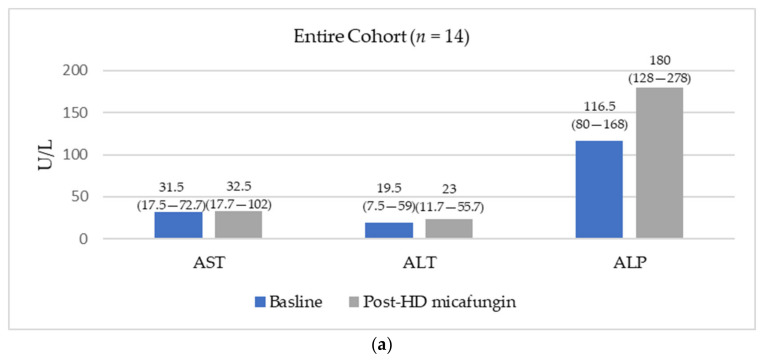

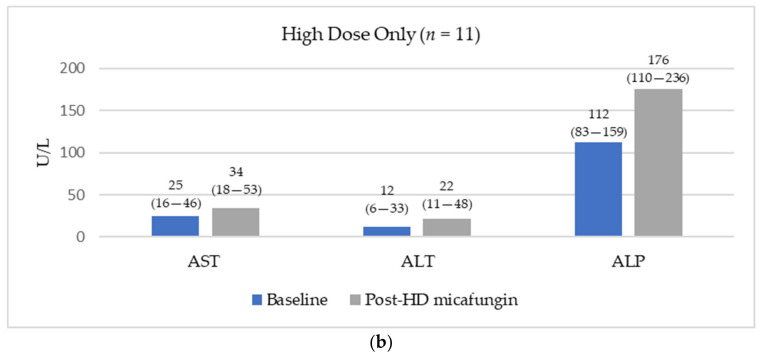
Median (interquartile range) baseline and post-HD micafungin AST, ALT, and ALP. Only includes patients with both baseline and post-HD micafungin laboratory data available. (**a**) Evaluates AST, ALT, and ALP trends among the entire cohort with available data; (**b**) evaluates AST, ALP, and ALP trends among patients that received HD micafungin for the entirety of their echinocandin course and had available data.

Baseline and post-HD micafungin AST, ALT, and ALP among patients with available laboratory data are displayed in Figure 1. Laboratory data were available for 14 encounters among the entire cohort (14/23, 60.9%) and 11 encounters among patients that received HD micafungin only (11/15, 73.3%). Median baseline AST, ALT, and ALP were WNL. For the entire cohort, AST and ALT remained stable. Among patients that received HD micafungin for their entire echinocandin course, AST and ALT trended upward by the end of HD micafungin treatment, but remained WNL. Alkaline phosphatase trended upward following HD micafungin (modestly above the upper limit of normal) for the entire cohort. A similar upward trend was seen among patients that received HD micafungin for their entire echinocandin course. Baseline platelets were WNL and remained stable following HD micafungin. Patients experienced nausea, vomiting, and diarrhea in seven (30.4%), six (26.1%), and three (13%) encounters, respectively. There were no reports of headache, rash, phlebitis, or infusion reactions. 

### 3.5. Clinical Outcomes

Overall, the median LOS and ICU LOS were 27 days (IQR 11–34) and 20 days (IQR 5.5–38.7), respectively. In a subgroup analysis of patients that received HD micafungin only, no differences in median LOS and ICU LOS were observed. Table 3 displays clinical outcome data among the entire cohort and among a subgroup of patients that received HD micafungin for the entirety of their echinocandin course. A total of four deaths occurred (17.4%). Table S1 provides descriptive comparisons of select demographic, clinical, and microbiologic data among patients that died and patients that survived. Patients that died were predominantly young, Hispanic males who were obese and/or critically ill. One patient died as a result of a discontinuation of all treatment and pursuit of comfort care measures only. The remaining three patients died following cardiac arrest while receiving full treatment. All-cause readmission within 30 days occurred following four encounters (17.4%). No patients experienced recurrent infection.

## 4. Discussion

*Candida albicans* remains the leading causative species of IC infections in the US [2]. However, rates of infection due to *Candida albicans* are declining as rates of infection due to alternative species, especially *Candida glabrata*, are rising [2]. This is particularly concerning because rates of mortality secondary to IC due to *Candida glabrata* are significantly higher than those due to *Candida albicans* [2]. Interestingly, although *Candida glabrata* was the most commonly isolated species in our study, no deaths involving IC cases due to *Candida glabrata* were observed. Similarly, increasing age has been identified as an independent risk factor for mortality among patients with IC [17]. Patients in our study that died were uncharacteristically young, with a median age of 35.5 years old. This may indicate that the patients included in this study represent a minority population compared to the large majority of patients included in epidemiologic reviews.

Further, in our study, patients who received at least three doses of HD micafungin tolerated therapy well, with only mild elevations in ALP seen. This is in line with previously published literature exhibiting little to no effect on hepatic enzymes with doses up to 8 mg/kg [11]. Gastrointestinal upset was common among our patients; however, its attributability to micafungin administration could not be well evaluated due to the retrospective nature of this study. Regardless, these would be considered mild events and would not be expected to result in patient harm or discontinuation of micafungin.

Overall, median LOS was 27 days, which is longer than the median 21 days reported in a national epidemiologic review of more than 130,000 hospitalizations associated with IC from 2002 to 2012 [18]. This review was unable to evaluate patient weight or clinical status during hospitalization due to data being collected from a large, national database with limited information. This makes it difficult to compare the patients in our cohort to those included in this study. It is possible that an extended LOS was observed in our study due to the large proportions of obese and/or critically ill patients included.

All-cause, inpatient mortality was 17.4%. This is lower than the 22% mortality seen among patients with IC in the previously mentioned epidemiologic review [2]. Among patients with candidemia, all-cause mortality was 40%. This is higher than the 28% mortality observed in candidemic patients from the same review [2]. A recent single-center, retrospective cohort study compared clinical outcomes between obese (median BMI 36.3 kg/m^2^) and non-obese (median BMI 20.4 kg/m^2^) patients with candidemia [19]. Micafungin was used empirically for most patients, all of whom received the standard 100 mg daily dose. Results showed statistically significant increases in infection-related LOS (13 days versus 19 days) and duration of candidemia (5 days versus 6 days) among obese patients. Although not statistically significant, rates of in-hospital mortality were proportionally higher in obese patients (13.5% versus 21.4%) [19]. Overall rates of mortality in this study (16.3%) were lower than the mortality rates reported among patients with candidemia in the previously mentioned epidemiologic review (28%) [2]. This makes it difficult to interpret the increased rates of mortality observed among the obese patients included in our study.

Similarly, a large review of patients with confirmed candidemia admitted to ICUs across 76 countries observed mortality rates > 40% [20]. Mortality among critically ill patients is often multifactorial and this review did not specifically evaluate inadequate antifungal dosing as a contributing factor. However, in the setting of potentially reduced PTA among critically ill patients treated with conventional doses of micafungin, it is reasonable to consider the impact that current dosing strategies may have on these patients.

These studies suggest that LOS may be longer and rates of mortality may be higher among obese and/or critically ill patients. However, the results are difficult to interpret and compare to the findings from our study due to differences in the included patient populations, potentially differing baseline rates of mortality, and lack of relevant data. More data are needed on a larger scale to better identify a cause and effect relationship between micafungin dosing among obese and/or critically patients and its potential impact on clinical outcomes.

There were several limitations to this study. First, we had a limited sample size. Therefore, it is difficult to draw strong conclusions from the entire cohort as well as subgroup analyses. However, given the novelty of this dosing strategy, we reported all retrievable data. Second, doses of micafungin were chosen on a case by case basis at the discretion of the ID attending physician. Therefore, we cannot make strong conclusions or suggestions regarding the specific dosing regimens utilized in this study. Third, this was a retrospective study and the inherent biases associated with this study design are noted. There were limitations in recording certain clinically relevant laboratory data due to the retrospective nature of this study, limiting our ability to analyze certain outcomes. Of note, although we were able to determine if source control was attempted, we were unable to evaluate if source control was adequate or the time it took to achieve source control. Finally, this study lacked a comparator arm. Therefore, we cannot make any conclusions regarding clinical outcomes among obese and/or critically ill patients treated with conventional doses of micafungin versus HD micafungin.

This study demonstrated that the real-world utilization of HD micafungin was well tolerated and showed similar rates of overall mortality when compared to nationally reported rates. Future studies that further investigate the potential need for higher doses of micafungin in obese and/or critically ill patients with IC are necessary. If high doses are warranted in these patients, the optimal dose and regimen will need to be determined. Although previously mentioned Monte Carlo simulations [9,10] suggest that specific high doses would result in adequate target attainment for certain MICs among obese and/or critically ill patients, this will need to be confirmed by pharmacokinetic evaluations following the administration of HD micafungin to these patients. Lastly, studies comparing outcomes among patients treated with conventional doses and patients treated with high doses of micafungin will need to be performed to confirm clinical superiority.

## Figures and Tables

**Table 1:** Baseline demographics and clinical characteristics.

Characteristic	Encounters (*n* = 23)
Age (y), median (IQR)	55 (34–66)
Male gender, *n* (%)	17 (73.9)
Weight (kg), mean (SD)	120.9 (50.7)
BMI (kg/m^2^), median (IQR)	37.1 (28.8–48.9)
Underweight (BMI < 18.5), *n* (%)	1 (4.3)
Normal-weight (BMI 18.5–24.9), *n* (%)	2 (8.7)
Overweight (BMI 25–29.9), *n* (%)	4 (17.4)
Obese (BMI 30–39.9), *n* (%)	8 (34.8)
Morbidly obese (BMI ≥ 40), *n* (%)	8 (34.8)
Race/Ethnicity, *n* (%)	
Hispanic/Latino	12 (52.2)
White (non-Hispanic/Latino)	8 (34.8)
Black (non-Hispanic/Latino)	3 (13)
Common comorbid conditions, *n* (%)	
Diabetes mellitus	14 (60.9)
Moderate–severe chronic kidney disease	8 (34.8)
Heart failure	8 (34.8)
Peripheral vascular disease	7 (30.4)
Charlson comorbidity index, median (IQR)	3 (0–7)
Common risk factors, *n* (%)	
Presence of central line	14 (60.9)
Receiving total parenteral nutrition	8 (34.8)
GI perforation during admission	5 (21.7)
RRT, *n* (%)	8 (34.8)
Sepsis criteria, *n* (%)	
Sepsis	6 (26.1)
Septic shock	4 (17.4)
Not septic	8 (34.8)
Unable to calculate due to missing data	5 (21.7)
Admitted to ICU at time of micafungin initiation	14 (60.9)
APACHE II score, median (IQR) *	24 (17.7–31)
ID consult, *n* (%)	20 (87)

APACHE: acute physiology and chronic health evaluation; BMI: body mass index; GI: gastrointestinal; ICU: intensive care unit; ID: infectious diseases; IQR: interquartile range; RRT: renal replacement therapy; SD: standard deviation; y: years. * APACHE II scores were calculated using data available for 10 patients admitted to the ICU.

**Table 2:** Microbiology data.

Characteristic	Encounters (*n* = 23)
Candidemia, *n* (%)	10 (43.5)
Source of infection, *n* (%)	
Intra-abdominal	9 (39.1)
Central line	7 (30.4)
Bone/joint	5 (21.7)
Other	2 (8.7)
Etiology, *n* (%)	*n* = 31
*Candida glabrata*	12 (38.7)
*Candida albicans*	10 (32.3)
*Candida parapsilosis*	2 (6.5)
*Candida tropicalis*	2 (6.5)
*Candida lusitaniae*	2 (6.5)
Other *Candida* species	3 (9.7)
Micafungin MIC distribution (mg/L), *n* (%)	*n* = 17
≤0.008	4 (23.5)
0.015	6 (35.3)
0.03	3 (17.6)
0.06	2 (11.8)
0.12	1 (5.9)
1	1 (5.9)
Micafungin MIC interpretation *, *n* (%)	*n* = 17
Susceptible	15 (88.2)
No interpretation	2 (11.8)
Persistently positive cultures	6 (26.1)

MIC: minimum inhibitory concentration; * Per CLSI M60 (June 2020) breakpoints.

**Table 3:** Micafungin Treatment data and clinical outcomes among the entire study cohort and a subgroup of patients that received high-dose micafungin for the entirety of their echinocandin course.

Characteristic	Entire Cohort (*n* = 23)	HD Only (*n* = 15)
ADD (mg), median (IQR)	300 (275–400)	300 (300–409.1)
ADD (mg/kg), median (IQR)	2.84 (2.4–3.9)	2.8 (2.3–3.6)
Micafungin duration (d), median (IQR)	11 (6–16)	11 (6-19)
LOS (d), median (IQR)	27 (11–45)	25 (11–45)
ICU LOS (d), median (IQR)	20 (5.5–38.7)	23.5 (5–46)
All-cause inpatient mortality, *n* (%)	4 (17.4)	3 (20)

ADD: average daily dose; d: days; HD: high dose; ICU: intensive care unit; IQR: interquartile range; LOS: length of stay.

## Data Availability

The data presented in this study are available on request from the corresponding author. The data are not publicly available due to privacy and ethical reasons.

## Supplementary Materials

The following are available online at https://www.mdpi.com/article/10.3390/tropicalmed7020023/s1, Table S1: Select baseline demographics, clinical characteristics, microbiology, and treatment data among patients that died and patients that survived.
