# Supplementary material for: Characterizing Safety and Clinical Outcomes Associated with High-Dose Micafungin Utilization in Patients with Proven Invasive Candidiasis
Source: Trop Med Infect Dis. 2022 Feb 3;7(2):23. doi: 10.3390/tropicalmed7020023 (PMC8878997; doi:10.3390/tropicalmed7020023)
Supplement: Supplementary file 1 [file tropicalmed-07-00023-s001.zip › tropicalmed-1558203-supplementary.pdf]

Tropical Medicine and Infectious Disease

MDPI

Brief Report

Characterizing Safety and Clinical Outcomes Associated with High-Dose Micafungin Utilization in Patients with Proven Invasive Candidiasis

Victoria C. Grant, Kenneth Nguyen, Sasha Rodriguez, Anna Y. Zhou, Jacinda C. Abdul-Mutakabbir and Karen K. Tan

Table S1. Select Baseline Demographics, Clinical Characteristics, Microbiology and Treatment Data Among Patients That Died and Patients That Survived.

Characteristic	Died (n = 4)	Survived (n = 19)
Age (y), median (IQR)	35.5 (31.7–39.2)	61 (34–67)
Male gender, n (%)	4 (100)	13 (68.4%)
Race, n (%)		
Hispanic/Latino	3 (75)	9 (47.4)
White (non-Hispanic/Latino)	1 (25)	7 (36.8)
Black (non-Hispanic/Latino)	0	3 (15.8)
Obese/morbidly obese, n (%)	3 (75)	13 (68.4)
Sepsis criteria, n (%)		
Sepsis	1 (25)	5 (26.3)
Septic Shock	2 (50)	2 (10.5)
Not septic	0	8 (42.1)
Unable to calculate due to missing data	1 (25)	4 (21.1)
ICU admission at time of micafungin initiation	4 (100)	10 (52.6)
APACHE II score, median (IQR)*	28 (13-31)	24 (18-31)
Candidemia, n (%)	4 (100)	6 (31.6)
Common sources, n (%)		
Central line	2 (50)	5 (26.3)
Intra-abdominal	1 (25)	8 (42.1)
Bone/joint	1 (25)	4 (21.1)
Received HD micafungin for entirety of echinocandin course, n (%)	3 (75)	12 (63.2)

APACHE: acute physiology and chronic health evaluation; HD: high dose; ICU: intensive care unit; IQR: interquartile range; y: years. * APACHE II scores were calculated using data from 3 and 7 patients that died and survived, respectively; the remaining patients admitted to the ICU could not have APACHE II scores calculated due to missing data.
